# Supplementary material for: Nutrients and non-essential metals in darkibor kale grown at urban and rural farms: A pilot study
Source: PLoS One. 2024 Apr 16;19(4):e0296840. doi: 10.1371/journal.pone.0296840 (PMC11020932; doi:10.1371/journal.pone.0296840)
Supplement: S1 File — (PDF) [file pone.0296840.s002.pdf]

Supporting Information

For *Nutrients and Non-Essential Metals in Darkibor Kale Grown at Urban and Rural Farms: A Pilot Study*. Brent F. Kim, Sara N. Lupolt, Raychel E. Santo, Grace Bachman, Xudong Zhu, Tianbao Yang, Naomi K. Fukagawa, Matthew L. Richardson, Carrie Green, Katherine M. Phillips, Keeve E. Nachman.

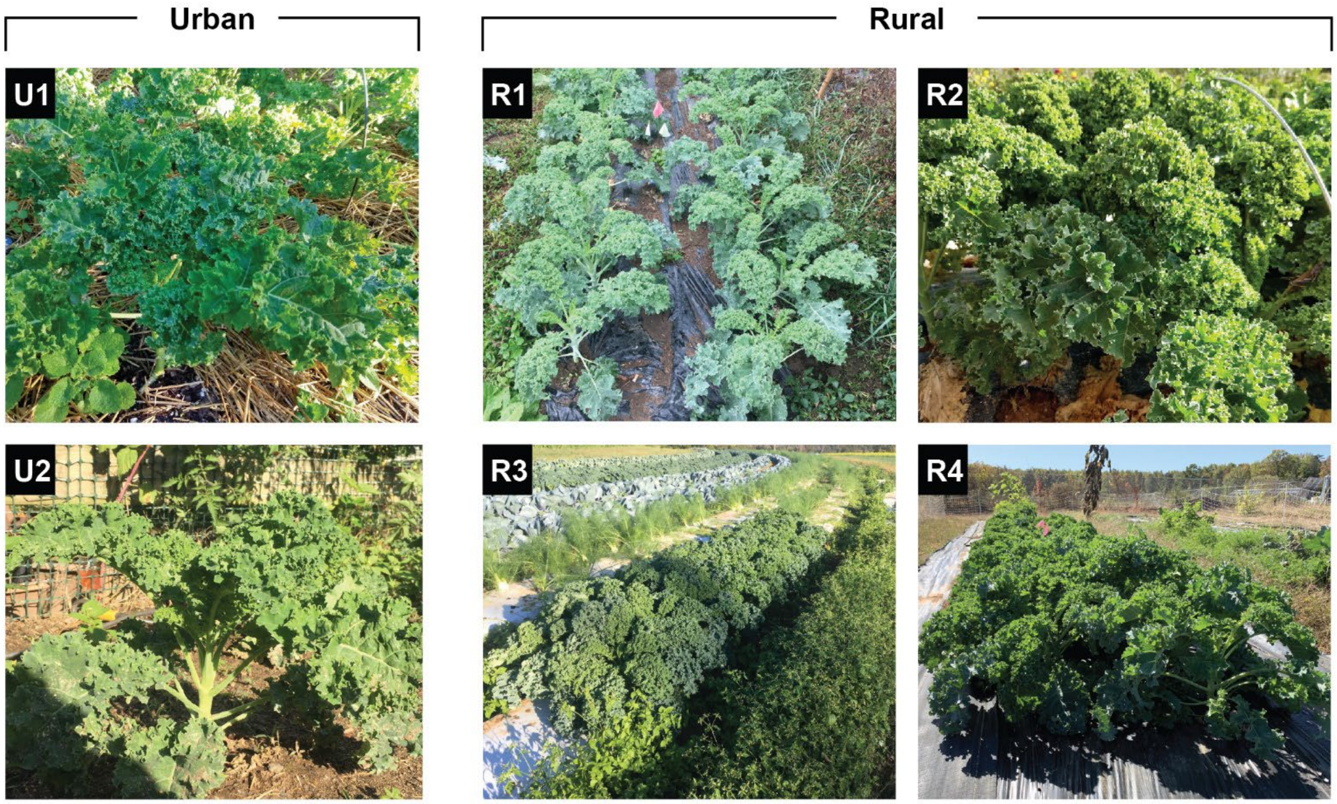

Fig S1. Photos of cultivated kale, by farm.

Site identifiers with “U” represent urban farms; identifiers with “R” represent rural farms.

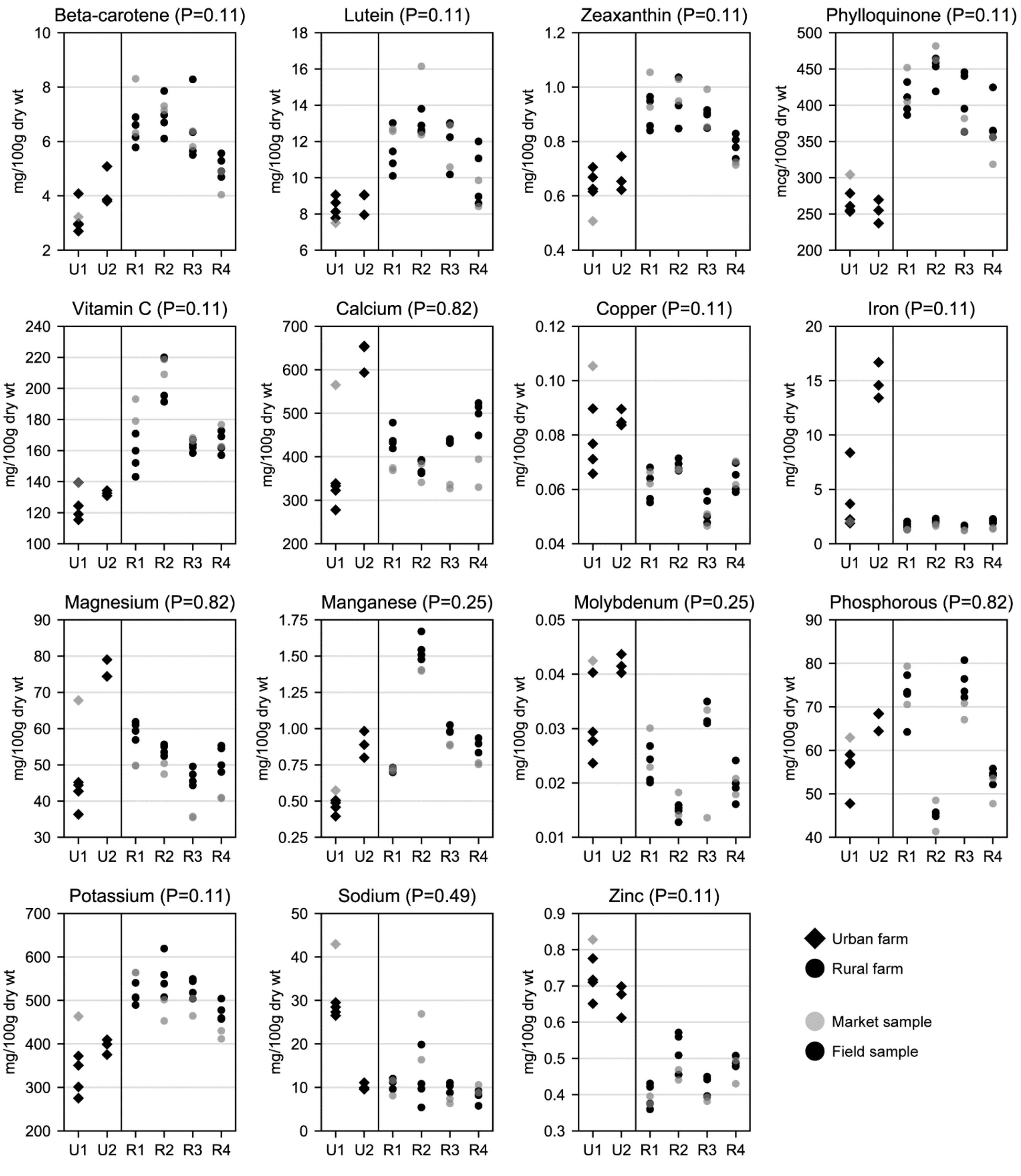

Fig S2. Dry weight concentrations of carotenoids, vitamins, and nutritional elements by farm, farm type (urban vs. rural), and sampling location (field vs. market).

Each dot represents one kale sample. Site identifiers (x-axis) with "U" represent urban farms, identifiers with "R" represent rural farms. P values are from Mann-Whitney U tests comparing field samples from urban and rural farms, using the mean value from each farm (N=6).

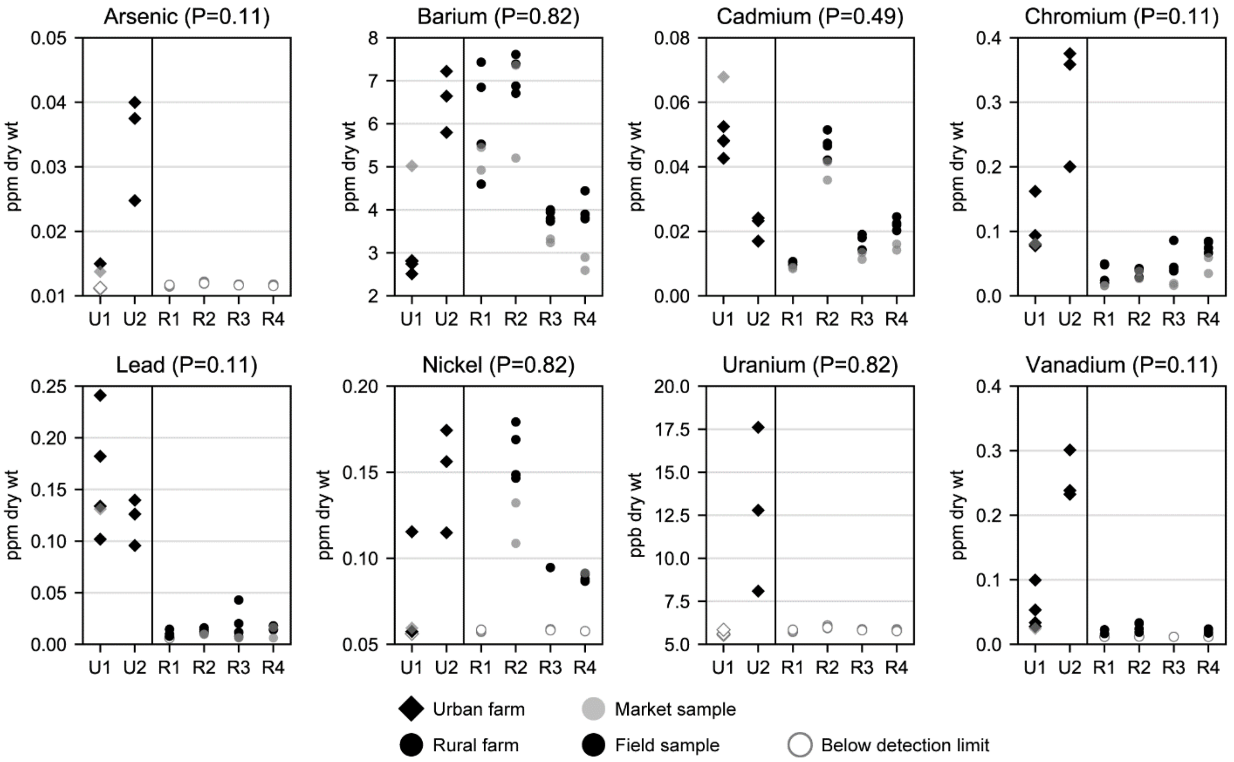

Fig S3. Dry weight concentrations of non-essential metals by farm, farm type (urban vs. rural), and sampling location (field vs. market).

Each dot represents one kale sample. Site identifiers (x-axis) with "U" represent urban farms, identifiers with "R" represent rural farms. P values are from Mann-Whitney U tests comparing field samples from urban and rural farms, using the mean value from each farm (N=6).

	Carotenoids & vitamins					Nutritional elements										Non-essential metals							
	β-carotene	Lutein	Zeaxanthin	Vit K1	Vit C	Ca	Cu	Fe	Mg	Mn	Mo	P	K	Na	Zn	As	Ba	Cd	Cr	Pb	Ni	U	V
Sample mass																							
Leaf:stem mass ratio			-	-			++	+								+			++			+	+
Moisture					-					- -			-										
Harvest to storage time							- -								-								

Negative correlation		No correlation	Positive correlation	
- -	-		+	++
P<0.01	P<0.05		P<0.05	P<0.01

Fig S4. Correlations between sample properties and dry weight analyte concentrations among mean field samples by farm.

Levels of statistical significance for Pearson's correlations between sample properties (mass, moisture, and time to freezing; table rows) and dry weight concentrations of nutrients and metals (table columns), in field samples, using the mean values from each farm (N=6).

		Carotenoids & vitamins					Nutritional elements										Non-essential metals							
		β-carotene	Lutein	Zeaxanthin	Vit K1	Vit C	Ca	Cu	Fe	Mg	Mn	Mo	P	K	Na	Zn	As	Ba	Cd	Cr	Pb	Ni	U	V
Carotenoids & vitamins	β-carotene		++	+++	++	+								+++		-					-			
	Lutein	++		++	++	+								++							-			
	Zeaxanthin	+++	++		++	+								++		-					-			
	Vit K1	++	++	++		+								++		-					- -			
	Vit C	+	+	+	+						+			+										
Nutritional elements	Ca									+							+						+	
	Cu								+							+				+				+
	Fe							+									+++			+++			+++	+++
	Mg						+										+						+	+
	Mn					+																		
	Mo																							
	P																		-					
	K	+++	++	++	++	+										-					- -			
	Na																							
	Zn	-		-	-			+						-							++			
Non-essential metals	As						+		+++	+										++			+++	+++
	Ba																							
	Cd												-											
	Cr							+	+++								++						++	+++
	Pb	-	-	-	- -									- -		++								
	Ni																							
	U						+		+++	+							+++			++				+++
	V							+	+++	+							+++			+++			+++	

Negative correlation			No correlation	Positive correlation		
- - -	- -	-		+	++	+++
P<0.001	P<0.01	P<0.05		P<0.05	P<0.01	P<0.001

Fig S5. Correlations between dry weight analyte concentrations among mean field samples by farm.

Levels of statistical significance for Pearson's correlations between mean dry weight concentrations of nutrients and metals in field samples, using the mean values from each farm (N=6).

				Carotenoids & vitamins					Nutritional elements										Non-essential metals							
Growing practice (number of farms using practice)	Sample mass	Leaf:stem ratio	Moisture	β-carotene	Lutein	Zeaxanthin	Vit K1	Vit C	Ca	Cu	Fe	Mg	Mn	Mo	P	K	Na	Zn	As	Ba	Cd	Cr	Pb	Ni	U	V
USDA Organic before planting (1)																										
Soil quality test before planting (4)			-										+												+	
Soil metals test before planting (3)		+		-	-	-	-									-						+				
Used pesticides before planting (4)			-										+												+	
Used pesticides after planting (4)									+								-				-					
Applied soil amendments >1x/year before planting (2)												+														
Applied feather meal before and/or after planting (2)			-	+	+		+						+			+										
Applied soil amendments after planting (5)																										
Used drip irrigation after planting (4)	+	-		+	+	+	+	+		-	-					+		-	-			-	-			-

Significant difference, mean lower among farms using practice: - (P<0.05)

No difference between farms using practice vs. not

Significant difference, mean higher among farms using practice: + (P<0.05)

Fig S6. Differences in mass, moisture, and dry weight analyte concentrations by growing practice among mean field samples by farm.

Levels of statistical significance for Mann-Whitney U tests (N=6) comparing mean mass, moisture, and dry weight concentrations of nutrients and metals in field samples (table columns) from farms that used a growing practice vs. those that did not (table rows). Parenthesized values indicate the number of farms, out of six, following each growing practice.

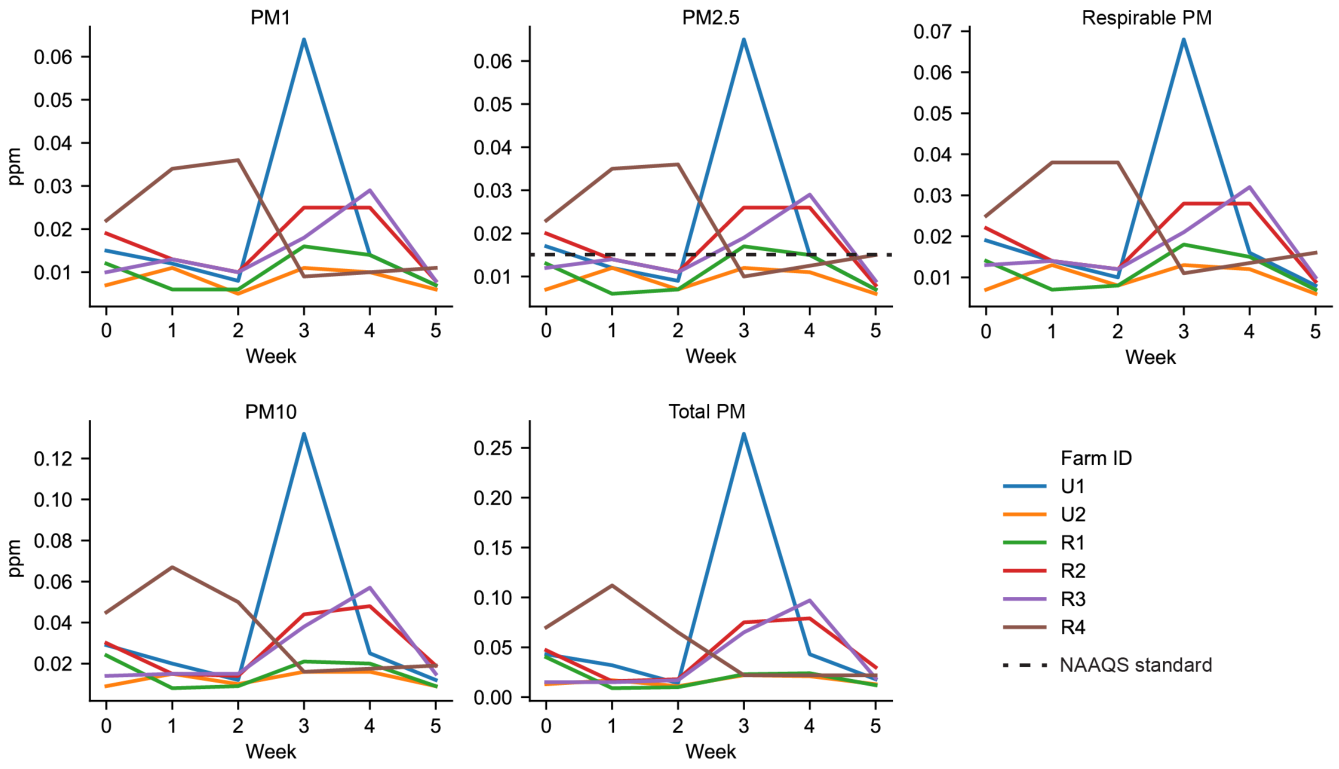

Fig S7. Particulate matter concentrations by farm.

Concentrations reported in parts per million (mg/m^3) for six farms over six weekly visits. Site identifiers with “U” represent urban farms; identifiers with “R” represent rural farms.

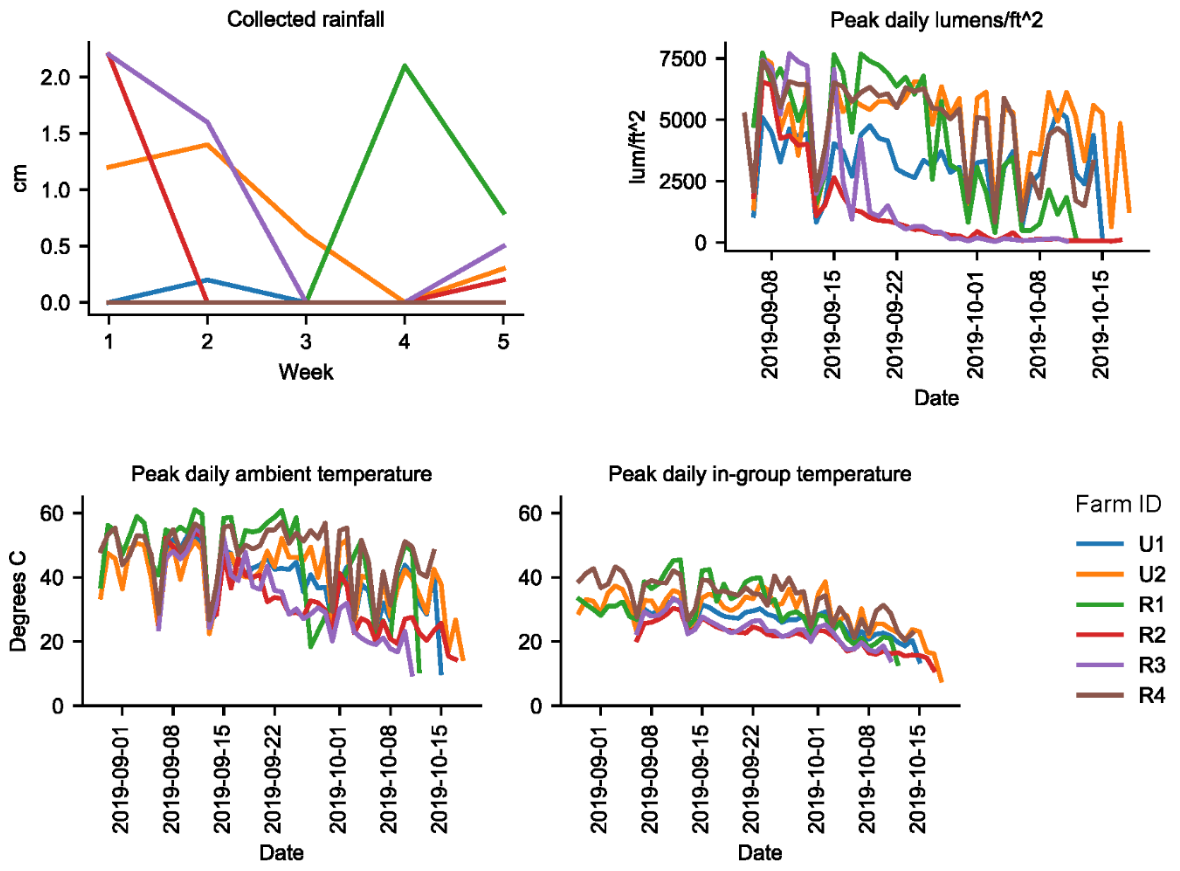

Fig S8. Rainfall, light, and temperature by farm.

Site identifiers with “U” represent urban farms; identifiers with “R” represent rural farms.

Baseline Site Questionnaire

Site name: ______________________________________ Site no: _________________

Address: ____

Date Collected: _______________

Name of person who completed survey: ___________________________________

- If different, who is the permanent site contact? _______________________________

SITE HISTORY

To your knowledge, what year was this site established? ________________

How was this land used before it became a farm (if you know)?

____

____

PLOT HISTORY

In the previous three years, what was grown in this area (where study kale will be grown)?

____

PREVIOUS KALE HARVESTS

How many years have you grown kale on your farm? ________________

Which varieties of kale do you typically grow?

____

The following questions are going to ask about your typical kale growing practices.

- ☐ How far apart do you space the:
  - kale plants? ________________
  - rows? ________________

☐ Do you intercrop the kale with anything else? Yes No

- If yes, which crops?

  ____

☐ Do you cover the kale with a tunnel or keep in the open air?

  ☐ high tunnel
  ☐ low tunnel
  ☐ open air

☐ Do you use cover crops during the winter? Yes No

- If yes, which crops?

  ____

- Do you rotate crop locations each year? Yes No

Which month(s) do you typically plant kale?

- Direct sow seeds: ____

  OR

- Plant seeds to grow seedlings: _____________________________________

- Plant seedlings in ground: ____

This year (not counting any kale produced via this study) how much kale do you anticipate growing (including both spring and fall plantings)?

In pounds __________

In sq ft or rows __________

GROWING PRACTICES

Is your farm currently certified USDA organic?

☐ Yes. Since when? __________________

☐ No. Has your farm ever been certified organic? If yes, when?

____

IRRIGATION WATER

What irrigation water sources do you use? Check all that apply.

- o Municipal water
- o Well water. If so, do you have a softener/treatment system? ______________________
- o Rain barrel
- o Other: _____________________
- o None
- o Don't know

How frequently do you water your crops?

- o More than once a day
- o Once a day
- o Once every two days
- o Twice a week
- o Once a week
- o Other: _____________________________

CONTAMINANT TESTING

Have you tested your soil for heavy metals and/or other contaminants? Yes No

- If yes, when was your most recent test? ___________________

- If yes, which metals did you test for?
  - ☐ Lead
  - ☐ Arsenic
  - ☐ Cadmium
  - ☐ Others: ____

- If yes, where did you send the sample(s) to be analyzed?

  ____

- If yes, would you be willing to share the results of those tests? Yes No

- If yes, how frequently do you test your soil for each contaminant?

  ____

SOIL QUALITY

Have you tested the quality of your soil (e.g., nutrients, fertility, pH)? Yes No

- If yes, when was your most recent test? __________________
- If yes, how did you test the soil (e.g. kit, agriculture or extension laboratory)?

  ____

- If yes, would you be willing to share the results of those tests? Yes No
- If yes, how frequently do you test your soil for each element?

  ____

SOIL AMENDMENTS
Have you applied any soil amendments (including chemical fertilizers, manure, compost) to the site this growing season?

☐ Yes
  - Which ones? ____
    - If compost, please specify the type or source of compost:

      ____

  - How much did you apply? _____________________________________
  - How often? ____________________________________

☐ No

PEST MANAGEMENT
Please list all pesticides (includes rodenticides, fungicides, insecticides) as well as natural deterrents applied to ANY crop this growing season.

1. ____
   a. How often did you apply this pesticide or deterrent? ____________________
   b. Approximately how much did you apply? ______________________________
2. ____
   a. How often did you apply this pesticide or deterrent? ____________________
   b. Approximately how much did you apply? ______________________________

3. ____

    a. How often did you apply this pesticide or deterrent? ____________________

    b. Approximately how much did you apply? ______________________________

4. ____

    a. How often did you apply this pesticide or deterrent? ____________________

    b. Approximately how much did you apply? ______________________________

5. ____

    a. How often did you apply this pesticide or deterrent? ____________________

    b. Approximately how much did you apply? ______________________________

FOOD SAFETY

Does your farm provide or encourage the use of gloves when doing any of the bellow activities?

- ○ Planting
    - Why or why not? ____
- ○ Weeding
    - Why or why not? ____
- ○ Watering
    - Why or why not? ____
- ○ Harvesting
    - Why or why not? ____

Does your farm have a designated location to eat lunch or rest? Yes No

Does your farm have….

☐ restrooms on site?

☐ handwashing facilities on site?

- ○ Do the handwashing facilities provide soap and water and/or hand sanitizers?
    - ○ Soap and water
    - ○ Hand sanitizers
- ○ Does your farm have any handwashing policies? If so, please describe:

  ____

Do any animals walk or roam in or near the fields where your kale is planted? (check all that apply)

- ☐ Birds
- ☐ Cats
- ☐ Chickens
- ☐ Dogs
- ☐ Foxes
- ☐ Groundhogs
- ☐ Raccoons
- ☐ Rodents
- ☐ Skunks
- ☐ Other: ______________________________________

Does your site have fencing?

- ☐ Yes. What type? ______________________________________

- ☐ No

HARVESTING, PROCESSING, AND DISTRIBUTION PRACTICES

How do you determine when your kale is ready to be harvested?

____

____

Which month(s) do you typically harvest kale?

____

How often do you harvest kale?

____

Please describe the procedure you use to harvest kale (e.g., do you use any tools, etc.)

____

____

Do you process kale on site for sale?

- ☐ Yes. How do you do it? ____
- ☐ No

How and where do you typically distribute or sell your kale? (name of location, city, address if known)

- ____
- ____
- ____
- ____
- ____
- ____

Besides kale, what other foods do you grow onsite?

____

____

____

How and where do you typically distribute or sell your other foods (if different from kale)?

- ____
- ____
- ____
