# Supplementary material for: Nutrients and non-essential metals in darkibor kale grown at urban and rural farms: A pilot study
Source: PLoS One. 2024 Apr 16;19(4):e0296840. doi: 10.1371/journal.pone.0296840 (PMC11020932; doi:10.1371/journal.pone.0296840)
Supplement: S2 File — (DOCX) [file pone.0296840.s003.docx]

Dear editors,

Please see our responses, below, to the requested changes dated 2023-12-28.

**1. Please ensure that the author list and affiliations are correct on the title page of your manuscript, and that your author contributions, competing interests, and financial disclosure are correct as listed below.**

Please revise the authors contributions as follows (with tracked changes):

Brent F Kim:

Data curation

Formal analysis

Investigation

Methodology

Software

Visualization

Writing – original draft

Writing – review & editing

Sara N. Lupolt:

Conceptualization

Investigation

Methodology

Project administration

Supervision

Writing – original draft

Writing – review & editing

Raychel E Santo:

Conceptualization

Investigation

Methodology

Project administration

Supervision

Writing – original draft

Writing – review & editing

Grace Bachman:

Investigation

Writing – review & editing

Xudong Zhu:

Investigation

Methodology

Writing – review & editing

Tianbao Yang:

Investigation

Methodology

Supervision

Writing – review & editing

Naomi K Fukagawa:

Conceptualization

Writing – review & editing

Matthew L Richardson:

Investigation

Writing – review & editing

Carrie Green:

Investigation

Methodology

Writing – review & editing

Katherine M Phillips:
Conceptualization

Funding acquisition

Investigation
Methodology
Validation
Writing – original draft
Writing – review & editing

Keeve E Nachman:

Conceptualization

Funding acquisition

Supervision

Writing – review & editing

**2. We note that the funders listed in your submission's Funding Information do not match the information given in your Financial Disclosure statement.**

The revised list of funders is as follows, with tracked changes:

- Agricultural Research Service, cooperative agreement 58-8040-8-018, Katherine M Phillips
- Agricultural Research Service, cooperative agreement 58-8040-8-021, Keeve E Nachman
- Johns Hopkins 21st Century Cities Initiative, dissertation grant, Sara Lupolt
- Johns Hopkins Education and Research Center for Occupational Safety and Health, supported by the National Institute for Occupational Safety and Health and the US Department of Agriculture Northeast Sustainable Agriculture Research and Education Program, pilot award GNE 19-209, Sara N. Lupolt
- Center for a Livable Future, Johns Hopkins Bloomberg School of Public Health, Lerner Fellowship, Sara N. Lupolt
- Johns Hopkins Institute for Clinical and Translational Research, National Center for Advancing Translational Sciences, UL1 TR003098, support for statistical consulting

The revised funding statement is as follows, with tracked changes; note this was included in our prior revisions submitted in early December, but the version shown in the list of revisions dated December 28 was still showing an earlier version, so we’re including it here again:

This work was supported by cooperative agreement 58-8040-8-018 between the US Department of Agriculture Agricultural Research Service (USDA ARS, <https://www.ars.usda.gov/>) and Virginia Tech, awarded to KP; and by cooperative agreement 58-8040-8-021 between the USDA ARS and Johns Hopkins University, awarded to KN. SL was supported by a dissertation grant from the Johns Hopkins 21st Century Cities Initiative (<https://21cc.jhu.edu/>); a pilot award from the Johns Hopkins Education and Research Center for Occupational Safety and Health (<https://publichealth.jhu.edu/johns-hopkins-education-and-research-center-for-occupational-safety-and-health>), supported by the National Institute for Occupational Safety and Health and the USDA Northeast Sustainable Agriculture Research and Education Program (GNE 19-209); and a Johns Hopkins Center for a Livable Future-Lerner Fellowship (<https://clf.jhsph.edu/education/clf-lerner-fellowships>). Support for statistical consulting was made possible by The Johns Hopkins Institute for Clinical and Translational Research (<https://ictr.johnshopkins.edu/>), funded in part by [Grant Number UL1 TR003098] the National Center for Advancing Translational Sciences, a component of the National Institutes of Health (NIH) and the NIH Roadmap for Medical Research. Because this was a cooperative agreement, three USDA ARS staff members (NF, CG, and TY) were involved in helping with study design, data collection and analysis, and preparation of the manuscript. All other funders had no role in study design, data collection and analysis, decision to publish, or preparation of the manuscript.

**3. Please rename your Supporting Information files to match what is written in your Supporting Information Caption located at the end of your manuscript.**

We have revised the filenames accordingly.

**4. In the text, cite the reference number in square brackets (e.g., “We used the techniques developed by our colleagues [19] to analyze the data”).**

We have revised in-text references accordingly.

**5. To prevent production delays, we recommend using the Author Formatting Checklist to confirm that your paper meets PLOS ONE's typesetting requirements for References, Tables, and Figures: http://journals.plos.org/plosone/s/file?id=c819/plos-one-author-formatting-checklist.docx.**

We were unable to download the checklist: “insecure download blocked”

**6. To ensure your figures meet our technical requirements, please run each figure included in your submission files through the PACE tool: https://pacev2.apexcovantage.com/. PACE will assess whether your figures meet our technical requirements and will fix the figure(s) or identify any problem(s) that cannot be automatically fixed. It can also convert figures to TIFF format, resize, and rename figures to meet our naming conventions.**

We ran the figures through PACE and have uploaded the revised versions.

Best regards,

Keeve Nachman, PhD, MHS
Robert S. Lawrence Associate Professor and Associate Chair of Environmental Health & Engineering
Johns Hopkins Bloomberg School of Public Health
Associate Director, Johns Hopkins Center for a Livable Future
Co-Director, Johns Hopkins Risk Sciences and Public Policy Institute
*(for the authorship team)*
