# Supplementary material for: Nutrients and non-essential metals in darkibor kale grown at urban and rural farms: A pilot study
Source: PLoS One. 2024 Apr 16;19(4):e0296840. doi: 10.1371/journal.pone.0296840 (PMC11020932; doi:10.1371/journal.pone.0296840)
Supplement: Attachment — Submitted filename: PONE-D-23-29655-Nutrients and Non-Essential Metals in Darkibor Kale Grown at Urban and Rural Farms.pdf [file pone.0296840.s004.pdf]

PONE-D-23-29655-Nutrients and Non-Essential Metals in Darkibor Kale Grown at Urban and Rural Farms: A Pilot Study

The words “Pilot study” may not be relevant in the publications.

Abstract

Lines 32-34 “In this pilot study we explored potential differences in nutritional elements, non-essential metals, and select health-relevant nutrients in Darkibor kale grown at urban versus rural farms”

What is the meaning of “pilot study” in this context?

What do authors mean by “Darkibor kale”- I guess Darkibor is a local name.

Lines 35-37 “Although small sample sizes precluded statistical significance for some analyses, we identified non-significant but potentially meaningful differences in concentrations of several components between urban and rural kale samples”

Clarify the meaning of “small sample sizes precluded statistical significance for some analyses’

The statements like “we identified non-significant but potentially meaningful differences…” This is quite confusing. Please present direct and explicit information, and given that this is an abstract, then should use catchy statements.

Lines 38-41 “Some urban-rural differences were more pronounced than those identified in prior research, although for some elements, variance within **urban and rural farms** was greater than variance between **urban and rural farms**”

Please rephrase the statement. See the bolded section.

Line 45: Introduction

This section was fairly well written though there were no compelling reasons to warrant research in Urban and Rural set up. For instance, are there more fumes as a result of vehicles and hence more heavy metal in urban areas? Let the background show previous work be reviewed showing the critical dissimilarities and find the gap that this work intended to fill. Please also clearly show the objective(s) of study and hypothesis/hypotheses (these should come out at the end of the section).

Line 102: Materials and Methods

This section is quite mixed up and quite confusing. This was supposed to be experimental research. Whereas in rural areas there were four (4) experimental farms, there were only two experimental farms in the rural area. Why this differences? My guess would be that the farms formed part of replications.

The experimental design is not shown but even more fundamental is that the replication does not comes out. For the rural areas, we assume that the four farms represent 4 replicates; which make lots of sense. However, the same cannot be said about the urban area. The two farms cannot be considered as a sufficient replication as this is not analysable.

The authors did not take initial soil samples before the start of the experiments. In that case it is not possible to speculate the possible causes in differences in kales grown in the urban areas compared to rural areas.

Line 103 Farm selection

It is not clear why this is being reported in the manuscript. I fully acknowledge the effort made in the identification of the farms, but the information is not necessary for the purposes of this manuscript. The authors can explain how the population in the rural and urban affect the mineral elements in the soil and hence uptake by the plant varieties.

Line 114 Kale seedling and planting

This subtitle is confusing – unless there were two sets where there was direct planting and transplanting of the seedlings. Can the authors clarify this?

Line 117 There is need to clarify the statement "The **seeds were sown in seedling** starting trays filled with Fafard growing mix" – see the bolded words

Lines 125-126 "in configurations most suitable for the location, e.g., **1x24, 2x12, 3x8 or 4x6**" – the authors need to clarify the spacing as shown in bold above. What these spacing/numbers stand for?

Line 130 Surveys on farm history and growing practices

This section may not be relevant unless during the process (survey), soil samples were picked and analysis made to check the chemical composition (including essential elements, beneficial elements, non-essential or toxic heavy metals and pesticides). Otherwise the way the section is presented, I do not find its relevance, but the authors can explain.

Line 161 "Kale sample processing and storage"

The authors need to justify the relevance of this section. Is it important that the vegetables be transported in refrigerated form yet tissue analysis (usual procedures) require heating at very high temperatures?

Line 180: Nutrient and non-essential metals analyses

In this section, there is need to clearly break down the methods- they cannot be lumped together- e.g. the methods for extraction of heavy metals is quite different compared for instance with the other essential elements. Method for P is totally different from say Ca and so they should be done as subsections. Then the vitamins and carotenoids are also determined using different protocols.

Still under this section, please explain the need for the vegetables being carried in ice box or freezer.

But looking at section starting from line 197- "analytical methods", then you will notice that there is no need for section on "**Nutrient and non-essential metals analyses**" above since section starting from line 197 is more comprehensive. Maybe have section as "Determination of minerals and vitamins in vegetables" to replace "analytical methods" and do away with "**Nutrient and non-essential metals analyses"**

However section in lines 209-213 need more elaboration – the analysis part of the metals is clear but the extraction methods not mentioned. A brief mention of the methods with citation of authors that the methods are adapted from can be quite useful.

Line 241: Data reporting and analyses

The contents should have been better be placed under "Results" section starting from section 286.

Why report in ppb instead of ppm? No wonder the metals were below the detectable limit leading to erroneous conclusion that the vegetables are safe (irrespective of whether cultivated in the urban or rural area (see abstract; line 41).

Line 255- there is a formula for conversion from fresh weight to dry weight- why not just dry the vegetables at a prescribed temperature till the weight is stable?

Line 286 Results

Line 287 Kale yields

In this section, reference is made of field samples and market samples – from materials and method section, such categorization was not evident. Can this be elaborated?

Table 1; These are raw data that have not been analysed and hence there is no point of presenting in this section.

Line 301: Farm history and growing practices

The section does fit under results – moreover, this would only be important if that history led to differences in factors like pH, organic carbon and nutrients as these would affect the availability of these elements to the crop. Can the authors explain the relevance of this section as far as the presentation of the results are concerned

Line 338; Kale sample processing and storage

This section does not belong to the "results" section. Is there any reason as to why this section is presented under "results" section?

Line 348: Analytical quality control

The authors present quite interesting results on different types of carotenoids. Unfortunately such differentiations/distinctions were not presented under "materials and methods' section.

Table 2: the heavy and essential elements are reported in terms of mg/100mg fresh weights. It is not common to report metal concentrations in fresh weights, instead dry weight is used. Any reasons for this exception?

And for the case when the concentration is in ppm fresh weight – why not mg/kg since we are dealing with solid particles and not liquid?

Note that here the reporting is in ppm yet in the materials and method the authors always made reference of ppb.

There is a column for “current mean- Urban” and “current mean, rural” – were these results analysed? Please use t-test to compare the two. But since the mean for urban had can only two replications, is it possible to have calculation using two replications?

In the figures, the symbols U1, U2, R1, R2, R3 and R4 are used yet there was no mention of these under “materials and method” section. It would have been polite for the authors to have figure captions where all these could be explained. Otherwise the way they are, it is not possible to interpret.

It is quite interesting that almost all the results show p-values more than 0.05 hence not significant. Is there possible reason? Could it be that calculation was not well done?

Discussion

Since the methodology needs serious reworking (a lot need to be cut out – especially the survey part and only remain with experimental components). And once the result section is more comprehensive and clear (cut out on survey and reduce the subsections under results; same with discussion). Once there is that kindly harmony, it is too premature to take a look at the discussion.
