# Supplementary material for: Nutrients and non-essential metals in darkibor kale grown at urban and rural farms: A pilot study
Source: PLoS One. 2024 Apr 16;19(4):e0296840. doi: 10.1371/journal.pone.0296840 (PMC11020932; doi:10.1371/journal.pone.0296840)
Supplement: Attachment — Submitted filename: Response to Reviewers.pdf [file pone.0296840.s005.pdf]

We are grateful for the reviewers' thoughtful and very thorough comments on the manuscript. We have made significant revisions in response to their suggestions, and believe the paper is much improved as a result. Please see our responses to comments below in bold.

ALL LINE NUMBERS BELOW REFER TO THE VERSION OF THE MANUSCRIPT WITH TRACKED CHANGES VISIBLE. The line numbers change when viewing the manuscript with tracked changes accepted.

Reviewer #1

General comment:

The pilot study discussed in this paper examines the nutritional elements, non-esSsential metals, and health-relevant nutrients in Darkibor kale grown in urban and rural settings. Despite its small sample size, the research reveals significant differences in kale composition between these two environments, which has important implications for the future of kale farming and nutrition.

Specific comments:

1. The paper effectively underscores the significance of kale as a nutrient-dense vegetable and its potential role in addressing the challenges posed by the climate crisis. This context is essential for understanding the importance of the study. What are the specific nutritional elements and non-essential metals that were found to exhibit differences between urban and rural-grown Darkibor kale in the study?

To clarify, the differences in metals/nutrients composition were *not* statistically significant between urban and rural environments. Per lines 430-436, at a sample size of N=6 (six mean values for each analyte, one per farm), it is not possible to obtain a P value ≤ 0.05 with a Mann Whitney U test.

We did, however, observe P values as low as 0.06 (for arsenic) and many other P values were low (P=0.11). To more clearly convey this, we have revised lines 451-455 in the Results to provide more detail, as follows:

> **"Small sample size (N=6 mean values for each analyte, one per farm) precluded the possibility of statistically significant differences in analyte concentrations between urban and rural field samples using Mann-Whitney U tests. However, P-values were as low as 0.06 for arsenic and 0.11 for all carotenoids and vitamins, copper, iron, potassium, zinc, lead, and vanadium (Figs 2-3)."**

2. The study's limitations in terms of sample size are acknowledged, but it does a commendable job in highlighting potentially meaningful differences in kale composition between urban and rural farms. However, more details about the sample size and methodology would be beneficial for readers to gauge the study's reliability.

Sample sizes are described in Table 1 and the "Kale yields" section of the Results. For some statistical tests comparing analyte concentrations between urban and rural kale, we had to use the mean concentration of that analyte for each of six farms. We have revised the text throughout to more clearly explain why the sample size for those tests was smaller than the 32 kale samples shown in Table 1. For example, lines 614-615 were revised as follows:

> **"…sample size (N=6 mean values for each analyte, one per farm) precluded the possibility of statistically significant differences between urban and rural field samples…"**

3. It's noteworthy that the research identifies higher concentrations of elements in kale from urban farms and elevated levels of carotenoids and vitamins in kale from rural farms. This specific insight adds value to our understanding of kale quality and can guide both urban and rural farming practices. The mention of variance within and between farm settings is an interesting observation that could lead to further research. Can you elaborate on the potential reasons behind the variations in kale composition between urban and rural farming environments, and how might these findings impact farming practices?

Line 624-668 in the discussion provide potential reasons why nutrient and metal composition may have been different between urban and rural kale. We have revised and elaborated on these points to say more about growing practices, e.g., irrigation and use of imported growing media. The revised sections read as follows:

> **"Urban farms' use of municipal water is unlikely to explain differences in kale metal concentrations, since the overwhelming majority of irrigation water samples from the SUH study were well below public health guidelines for nine metals (35). Since the rural farms in this study reported using drip irrigation and urban farms did not, moisture stress could partly explain the lower carotenoid, vitamin, and potassium concentrations in urban kale (Fig 6), which for potassium would be consistent with a prior study suggesting concentrations of some minerals in kale decrease as a result of moisture stress (19). Contrary to the prior study, however, in this study concentrations of copper and iron were *higher* among kale samples experiencing greater water stress (i.e., urban kale, on the hypothesis that use of drip irrigation alleviates water stress). Further research on kale could explore the effects of these and other factors or urban-rural differences, including by analyzing irrigation water samples as in Lupolt et al. (35)."**

> **"To mitigate soil quality and contamination concerns, farmers may grow crops in raised beds filled with imported growing media. Both urban farms in the current study reported using some compost, but it was unclear whether they used imported growing media exclusively, or if it was added to native soil. The overwhelming majority (95%, among the 100 farms that responded to questions about soil) of Baltimore's farms and gardens participating in SUH reported growing crops in at least some soil, compost, or mulch brought in from off-site, and two-thirds (69%) used raised beds (10). Media used to fill raised beds should be of good quality and not contaminated, although even the use of clean soils does not address the potential for soil recontamination via airborne deposition (31)."**

4. This paper sheds light on the nutritional composition of Darkibor kale grown in urban and rural settings, highlighting distinctions between the two. Despite the small sample size, the study presents valuable insights into kale quality and the factors contributing to these differences. Considering the importance of kale in addressing the climate crisis and food security, what future research or actions do you believe are necessary based on the insights gained from this pilot study?

In the discussion ("Study limitations and lessons learned"), we describe some challenges and lessons learned about survey questions and the real-world challenges faced by urban farmers, and the conclusion has some recommendations for future research. We have revised and expanded the discussion slightly to make some of the recommendations more explicit, for example:

> **"…Given the popularity of farmers markets, future research could explore the potential for nutrient loss between harvest and point of sale, with an eye toward helping producers make decisions about how best to preserve nutrient density."**
>
> **"…Future studies on urban agriculture should plan and account for these realities, while exploring policy interventions to help address them."**

Constructive feedback:

The paper could be strengthened by providing a more comprehensive explanation of the methodology employed in the study. Additionally, including data on the actual concentrations of the identified elements and nutrients would help readers better grasp the significance of the findings.

We agree with the reviewer's comment below that it is helpful, in the context of public health guidelines, to refer to specific concentrations in the text. See revisions in response to the next comment below.

There are a large number of quantitative values to report (23 analytes for each of 32 samples, both in fresh and dry weight concentrations: 23 x 32 x 2 = 1,472 values to report). For the sake of brevity, we did not want to burden readers with too many in-text references to quantitative values, especially given all of this information is provided, in detail, in the figures and tables:

- **Mean urban and mean rural concentrations, for each analyte, are provided in Table 2.**
- **Fresh weight analyte concentrations for individual kale samples are displayed in Figures 2 and 3.**
- **Dry weight analyte concentrations for individual kale samples are displayed in Figures S2 and S2.**
- **Fresh and dry weight concentrations for individual kale samples are reported in Table S2 and summarized (e.g., means, ranges, IQR) in Tables S3 (by farm) and S4 (over all samples).**

While the study rightly mentions that concentrations of metals remained below public health guidelines, it would be beneficial to explicitly state the actual concentrations and compare them to those guidelines for clarity.

We have revised lines 461-468 in the Results to explicitly state mean concentrations when comparing them to public health guidelines, as follows:

> **"…lead and cadmium concentrations for all samples, including urban (mean Pb: 0.13 ppm, mead Cd: 0.03 ppm) and rural (mean Pb: 0.01 ppm, mean Cd: 0.02 ppm) field samples, were below the maximum levels (Pb: 0.3 ppm, Cd: 0.2 ppm; Fig 3) specified by the Food and Agriculture Organization of the United Nations (FAO) and the World Health Organization (WHO) (60)."**

Summary:

The significance of kale as a climate-resilient crop is well articulated, and the study motivates further investigation in this area, which is crucial given the increasing popularity of this nutrient-dense vegetable. However, to improve its impact, the paper should provide more detailed information on the methodology and specific concentration levels of elements and nutrients.

Reviewer #2

The manuscript entitled "Nutrients and Non-Essential Metals in Darkibor Kale Grown at Urban and Rural Farms: A Pilot Study" has been reviewed. The manuscript is well written; however, before the final acceptance authors should execute the following minor corrections:

The abstract is too much descriptive. Please add some statistical findings.

We have greatly expanded the abstract to include additional details about the methodology, quantitative statistics, and several concluding statements about key conclusions:

> **"Kale is a nutrient-dense leafy vegetable associated with wide-ranging health benefits. It is tolerant of drought and temperature fluctuations, and could thus serve an increasingly important role in providing a safe and nutritious food supply during the climate crisis, while kale's ease of cultivation and ability to be grown in a wide range of soils make it a good fit for urban agriculture. In this pilot study we explored potential differences between kale grown at urban versus rural farms. We planted kale seedlings (Darkibor variety) at three urban and four rural farms in and around Baltimore City, Maryland, instructed farmers to cultivate them using their usual growing practices, harvested the kale from fields and points of distribution, and analyzed it for concentrations of carotenoids, vitamins C and K_1, ten nutritional elements, and eight non-essential metals. Although sample sizes for some analyses were in some cases too small to produce statistically significant results, we identified non-significant but potentially meaningful differences in concentrations of several components between urban and rural kale samples. Compared to urban samples, mean concentrations of carotenoids and vitamins were 22-38% higher in rural field samples. By contrast, mean concentrations for eight nutritional elements were higher in urban field samples by as much as 413% for iron. Compared to rural field samples, mean concentrations of nine non-essential metals were higher in urban samples, although lead and cadmium concentrations for all samples were below public health guidelines. Some urban-rural**

differences were more pronounced than those identified in prior research. For six elements, variance within urban and rural farms was greater than variance between urban and rural farms, suggesting urbanicity may not be the primary driver of some observed differences. For some nutrients, mean concentrations were higher than upper ranges reported in prior estimates, suggesting kale may have the potential to be more nutrient-dense than previously estimated. The nutritive and metals composition of this important crop, and the factors that influence it, merit continued investigation given its growing popularity."

2. Line 124, "Between 27-29 August" please mention the year.

We have made this change: "Between 27-29 August 2019…"

3. Line 132, "Supporting" should be "supporting".

Consistent with how we referred to it elsewhere, we changed "Supporting information" to "Supporting Information." Since this refers to the title of a document we felt title capitalization would be most appropriate.

We also realized the baseline survey was not included in the previous version of the Supporting Information, so we added it and attached a revised version.

Reviewer #3

This study explored potential differences in nutritional elements, non-essential metals, and certain health-relevant nutrients in Darkibor kale grown at urban versus rural farms. The objectives are clear. The selection of farms for planting and survey are good representatives. The sample harvesting and processing, the analyses of different parameters are well-described. The results collected are meaningful. Although there are constraints such as small sample sizes, which precluded statistical significance for some analyses, this study identified non-significant but potentially meaningful differences in in concentrations of several components between urban and rural kale samples. The paper is well-written. It can be accepted for publication in "PLOS ONE": after making the following revision:

1. Line 48: "According to …".

Good catch, we have made this change.

2. Line 126: "All plants were grown in open air, …" Is it possible to provide the growth conditions of different farm?

The section on "Farm history and growing practices" describes use of pesticides, irrigation, soil amendments, and other growing practices. Supplementary Figures S7 and S8 describe particular matter concentrations, rainfall, light intensity, and temperature, over time, for each farm. We did not collect any additional information about growing conditions.

3. Table 2: It would be better to include standard errors and statistical analysis for the means.

We have added standard errors to the means in Table 2. We have also added standard errors to the summary statistics provided in Tables S3 and S4.

4. Lines 419 - 421: "…contrast, with the exception of manganese and potassium, mean concentrations of nutritional elements were higher in urban field samples by as much as 413% for iron. Why? Was the lower iron concentration of kale from the rural resulting from iron deficiency?

The quoted text is from the Results section; we provide interpretation, discussion, and possible explanations for our findings in the Discussion section.

Urban kale samples were exceptionally high in iron, per line 602, "Iron concentrations were 374% higher in urban samples from this study compared to mean USDA values." In the discussion section on "Differences between urban and rural samples," we discuss factors that could explain urban-rural differences, e.g., sources of environmental contamination, differences in soil conditions, and use of raised beds and imported media. We have also expanded this discussion to say more about differences in water sources and irrigation methods, as follows:

> **"Urban farms' use of municipal water is unlikely to explain differences in kale metal concentrations, since the overwhelming majority of irrigation water samples from the SUH study were well below public health guidelines for nine metals (27). Since the rural farms in this study reported using drip irrigation and urban farms did not, moisture stress could partly explain the lower carotenoid, vitamin, and potassium concentrations in urban kale (Fig 6), which for potassium would be consistent with a prior study suggesting concentrations of some minerals in kale decrease as a result of moisture stress (19). Contrary to the prior study, however, in this study concentrations of copper and iron were *higher* among kale samples experiencing greater water stress (i.e., urban kale, on the hypothesis that use of drip irrigation alleviates water stress). Further research on kale could explore the effects of these and other factors or urban-rural differences, including by analyzing irrigation water samples as in Lupolt et al. (35)."**

5. Lines 468 – 471 "Similarly, concentrations of elements and metals in some cases positively correlated with one another, e.g., iron with copper, magnesium, arsenic, chromium, uranium, and vanadium ($P<0.05$). Potassium was positively correlated with carotenoids and vitamins, while lead and zinc were inversely correlated with most carotenoids and vitamins ($P<0.05$)." Why?

The quoted text is from the Results section; we provide interpretation, discussion, and possible explanations for our findings in the Discussion section. See response to comment 7 below.

6. Lines 470-471: "Potassium was positively correlated with carotenoids and vitamins, while lead and zinc were inversely correlated with most carotenoids and vitamins ($P<0.05$). Why? Any explanation with references to support?

The quoted text is from the Results section; we provide interpretation, discussion, and possible explanations for our findings in the Discussion section. See response to comment 7 below.

7. Lines 635-639: "By contrast, concentrations of lead and zinc were negatively correlated with concentrations of carotenoids and vitamins. Since urban farms were generally significantly lower in carotenoids and vitamins and higher in elements compared to rural farms, urbanicity—and the associated properties of urban and rural soils explained above—could be the confounders that explain these differences." From Table 2, kale grown in urban farm had much higher Zinc that rural farm. Why urban farms were generally significantly lower in carotenoids and vitamins? Were the lead and zinc concentrations in urban farm soil too high, which resulted in the reductions of carotenoids and vitamins?

This is an excellent question. We did not measure soil metals concentrations, so we can only hypothesize about the possible explanations. We revised this section slightly to incorporate more evidence from prior literature, as follows:

> **"By contrast, concentrations of lead and zinc were negatively correlated with concentrations of carotenoids and vitamins. For lead, this is consistent with prior evidence of an inverse association between heavy metal and vitamin concentrations in plants, in part due to oxidative stress (65)."**

Reviewer #4

PONE-D-23-29655-Nutrients and Non-Essential Metals in Darkibor Kale Grown at Urban and Rural Farms: A Pilot Study

The words "Pilot study" may not be relevant in the publications.

Abstract

Lines 32-34 "In this pilot study we explored potential differences in nutritional elements, non-essential metals, and select health-relevant nutrients in Darkibor kale grown at urban versus rural farms"

What is the meaning of "pilot study" in this context?

This was a small study to test research protocols and other research techniques. Our hope is to set the stage for a follow-up study that could explore our research questions with a larger sample size, incorporating lessons learned from this initial work.

What do authors mean by "Darkibor kale"- I guess Darkibor is a local name.

We have revised the language in the abstract to say "We planted kale seedlings (Darkibor variety) at three urban and four rural farms in and around Baltimore City…"

Lines 35-37 “Although small sample sizes precluded statistical significance for some analyses, we identified non-significant but potentially meaningful differences in concentrations of several components between urban and rural kale samples”

Clarify the meaning of “small sample sizes precluded statistical significance for some analyses’

The statements like “we identified non-significant but potentially meaningful differences…” This is quite confusing. Please present direct and explicit information, and given that this is an abstract, then should use catchy statements.

We have revised the language in the abstract as follows: “Although sample sizes for some analyses were in some cases too small to produce statistically significant results, we identified non-significant but potentially meaningful differences in concentrations.”

Following that sentence we added explicit detail to the abstract; please see our response to the next comment below.

Lines 38-41 “Some urban-rural differences were more pronounced than those identified in prior research, although for some elements, variance within urban and rural farms was greater than variance between urban and rural farms”

Please rephrase the statement. See the bolded section.

We have greatly expanded the abstract to include additional details about the methodology, quantitative statistics, and several concluding statements about key conclusions:

> **“Kale is a nutrient-dense leafy vegetable associated with wide-ranging health benefits. It is tolerant of drought and temperature fluctuations, and could thus serve an increasingly important role in providing a safe and nutritious food supply during the climate crisis, while kale’s ease of cultivation and ability to be grown in a wide range of soils make it a good fit for urban agriculture. In this pilot study we explored potential differences between kale grown at urban versus rural farms. We planted kale seedlings (Darkibor variety) at three urban and four rural farms in and around Baltimore City, Maryland, instructed farmers to cultivate them using their usual growing practices, harvested the kale from fields and points of distribution, and analyzed it for concentrations of carotenoids, vitamins C and K_1, ten nutritional elements, and eight non-essential metals. Although sample sizes for some analyses were in some cases too small to produce statistically significant results, we identified non-significant but potentially meaningful differences in concentrations of several components between urban and rural kale samples. Compared to urban samples, mean concentrations of carotenoids and vitamins were 22-38% higher in rural field samples. By contrast, mean concentrations for eight nutritional elements were higher in urban field samples by as much as 413% for iron. Compared to rural field samples, mean concentrations of nine non-essential metals were higher in urban samples, although lead and cadmium concentrations for all samples were below public health guidelines. Some urban-rural differences were more pronounced than those identified in prior research. For six**

elements, variance within urban and rural farms was greater than variance between urban and rural farms, suggesting urbanicity may not be the primary driver of some observed differences. For some nutrients, mean concentrations were higher than upper ranges reported in prior estimates, suggesting kale may have the potential to be more nutrient-dense than previously estimated. The nutritive and metals composition of this important crop, and the factors that influence it, merit continued investigation given its growing popularity."

Line 45: Introduction

This section was fairly well written though there were no compelling reasons to warrant research in Urban and Rural set up. For instance, are there more fumes as a result of vehicles and hence more heavy metal in urban areas? Let the background show previous work be reviewed showing the critical dissimilarities and find the gap that this work intended to fill. Please also clearly show the objective(s) of study and hypothesis/hypotheses (these should come out at the end of the section).

We have revised the last paragraph of the introduction to more clearly convey the study objectives, as follows:

> **"Building upon the existing body of evidence, we conducted a pilot study to assess levels of select health-relevant nutrients, nutritional elements, and non-essential metals in kale grown at two urban and four rural farms; and to gather data about site history, farming practices, environmental conditions, and other factors that might influence the properties of kale grown at those sites. The aim of the study was to explore the degree to which concentrations of these analytes might differ between urban and rural farms, and possible reasons for those differences…"**

We agree with the reviewer that it would be helpful to provide more background on urban contamination sources in the Introduction, so we have added lines 91-97 to the Introduction as follows, moving up and adapting some content that was previously in the Discussion:

> **"Environmental factors can also impact concentrations of non-essential metals and other harmful contaminants in kale. Urban areas, for example, often have high concentrations of industrial activity, waste incineration, building demolition, lead-based paint, vehicular emissions, tire wear, and other sources of harmful metals (27–31). Releases from these sources may contaminate growing soils via various environmental pathways, including runoff and airborne deposition (29,31), and subsequently be taken up by plant tissues. Uptake rates may vary widely by compound, plant species, and the part of the plant, e.g., roots vs. leaves (32–34)."**

Accordingly, to minimize redundancy, we have streamlined mention of soil contamination sources in the Discussion, consolidating it with another paragraph about soil fertility:

> **"Soil conditions could also explain urban-rural differences in kale. Urban soils may be subject to numerous factors that can decrease fertility, including compaction by buildings and heavy machinery which, in turn, can decrease porosity, suppress the**

> **activity of beneficial microbes, and inhibit the accumulation of soil organic carbon (63), all of which may affect the nutrient density of urban crops. Urban areas are also often associated with sources of soil contamination (27–31) which may be taken up by plant tissues or deposited on surfaces (see Introduction). We would not expect soil on the surfaces of kale plants to be a major factor in this study given samples were thoroughly washed, although soil resides may have remained in crevices of the leaves. Follow-up studies should account for these contamination pathways by analyzing soil samples, as in Lupolt et al. (35).”**

Line 102: Materials and Methods

This section is quite mixed up and quite confusing. This was supposed to be experimental research. Whereas in rural areas there were four (4) experimental farms, there were only two experimental farms in the rural area. Why this differences? My guess would be that the farms formed part of replications.

See response to the next comment below.

The experimental design is not shown but even more fundamental is that the replication does not comes out. For the rural areas, we assume that the four farms represent 4 replicates; which make lots of sense. However, the same cannot be said about the urban area. The two farms cannot be considered as a sufficient replication as this is not analysable.

We understand the reviewer’s concern about sample size. Our original research plan included a third urban farm, but a pest infestation eliminated most of their kale, leaving us with only two urban farms. Ideally, we would have had a larger sample size (i.e., more farms) but for the purpose of a small pilot study this was sufficient.

For the purpose of statistical comparisons (e.g., Mann Whitney U tests, described in lines 288-291 of the Methods), it is not necessary to have an equal number of urban and rural farms. We are unclear on the reviewer’s use of “replicates” as it refers to farms. In the context of our study, our understanding of the term “replicate” (or “duplicate”) refers to repeating the same analysis (e.g., vitamin C concentrations) on the same kale sample multiple times.

The authors did not take initial soil samples before the start of the experiments. In that case it is not possible to speculate the possible causes in differences in kales grown in the urban areas compared to rural areas.

We appreciate the reviewer’s interest in soil sampling. We agree soil sampling and analyses would have been very informative, but this was outside the scope of this study. We have added the following to the paragraph discussion soil contamination pathways (line 650):

> **“…Follow-up studies should account for these contamination pathways by analyzing soil samples, as in Lupolt et al. (35).”**

Line 103 Farm selection

It is not clear why this is being reported in the manuscript. I fully acknowledge the effort made in the identification of the farms, but the information is not necessary for the purposes of this manuscript. The authors can explain how the population in the rural and urban affect the mineral elements in the soil and hence uptake by the plant varieties.

We believe the methods by which we recruited participating farms should be included for the sake of transparency. The farm selection process may also have implications for the degree to which the study farms are representative of urban and rural farms as a whole, especially since the farms were not randomly chosen. Researchers seeking to conduct similar studies may also benefit from learning about our recruitment process.

Line 114 Kale seedling and planting

This subtitle is confusing – unless there were two sets where there was direct planting and transplanting of the seedlings. Can the authors clarify this?

The header refers to the process of growing the seedlings, then subsequently transplanting them at the farms. In the header and elsewhere in the methods, for greater clarity we have replaced the word "planting" with "transplanting."

Line 117 There is need to clarify the statement "The **seeds were sown in seedling** starting trays filled with Fafard growing mix" – see the bolded words

We are not sure what is unclear about this statement. "Sowing" is the process of planting seeds. The seeds were planted in seedling starting trays.

Lines 125-126 "in configurations most suitable for the location, e.g., 1x24, 2x12, 3x8 or 4x6" – the authors need to clarify the spacing as shown in bold above. What these spacing/numbers stand for?

We agree this was confusing, and have revised/corrected this part as follows:

> **"Between 27-29 August 2019, we planted 32 seedlings at each farm using stainless steel trowels at a spacing of 12 inches (30 cm) in an area determined by the farmer, in configurations most suitable for the location, e.g., 1 row of 32 seedlings, 2x16, or 4x8."**

Line 130 Surveys on farm history and growing practices

This section may not be relevant unless during the process (survey), soil samples were picked and analysis made to check the chemical composition (including essential elements, beneficial elements, non-essential or toxic heavy metals and pesticides). Otherwise the way the section is presented, I do not find its relevance, but the authors can explain.

We agree soil sampling and analyses would have been very informative, but this was outside the scope of this study.

The surveys were a relevant part of the study design, as they allowed us to gather data about farm characteristics, growing practices, time between harvest and distribution at markets, and other information that could help explain observed nutrient and metals concentrations and provide context for the results.

Line 161 "Kale sample processing and storage"

The authors need to justify the relevance of this section. Is it important that the vegetables be transported in refrigerated form yet tissue analysis (usual procedures) require heating at very high temperatures?

We have changed the name of the header from "Sample processing" to "Kale sample processing and storage." We agree this is title is clearer.

Vitamin C and carotenoids are susceptible to oxidation, thus we froze kale samples to inhibit this process.

As explained in the methods and associated reference citations, vitamin C was analyzed by validated methodology, including studies on stability of homogenized raw vegetables. The processing of the samples in liquid nitrogen, storage under residual nitrogen at -60C, and including of antioxidants in the extraction procedure (which is not conducted at elevated temperature) as well as the fact the both ascorbic acid and dehydroascorbic acid were quantified (= total ascorbic acid=vitamin C) accounts for the possibility of oxidation during analysis. (Statement added at lines 227-229).

Line 180: Nutrient and non-essential metals analyses

In this section, there is need to clearly break down the methods- they cannot be lumped together- e.g. the methods for extraction of heavy metals is quite different compared for instance with the other essential elements. Method for P is totally different from say Ca and so they should be done as subsections. Then the vitamins and carotenoids are also determined using different protocols.

All of the methods used have been previously reported in the references cited (including different citations for the nutritional elements and metals), so details are not repeated and only a brief description of methodology is noted (also see response below)

Still under this section, please explain the need for the vegetables being carried in ice box or freezer.

The kale was transported in a cooler to prevent subjecting different samples to different post-harvest conditions, since the study was on the effect of production conditions.

But looking at section starting from line 197- "analytical methods", then you will notice that there is no need for section on "Nutrient and non-essential metals analyses" above since section starting from line 197 is more comprehensive. Maybe have section as "Determination of minerals and vitamins in

vegetables" to replace "analytical methods" and do away with "Nutrient and non-essential metals analyses"

It's not immediately obvious from the subtle difference in font size, but "Nutrient and non-essential metals analyses" is a level 2 header, under which "Analytical methods" and "Analytical quality control" are level 3 sub-headers.

However section in lines 209-213 need more elaboration – the analysis part of the metals is clear but the extraction methods not mentioned. A brief mention of the methods with citation of authors that the methods are adapted from can be quite useful.

For the sake of brevity, citations for lines 209-213 (now 236-240) are already provided in lieu of repeating all of the methods in detail:

> **Nine elements (Ca, Cu, Fe, K, Na, Mn, Mg, P, and Zn) were analyzed by inductively coupled plasma emission spectroscopy (ICP) after digestion with concentrated hydrochloric acid (50). The remaining elements (As, Ba, Cd, Cr, Pb, Mo, Ni, U, V) were analyzed by ICP with mass spectrometry detection (ICP-MS) after digestion with concentrated nitric acid and water using a closed-vessel microwave digestion system (51,52).**
>
> **50. Association of Official Analytical Chemists International. Official Methods of Analysis, Method 984.27, 985.01,and 2011.14. Gaithersburg, MD, USA;**
>
> **51. Association of Official Analytical Chemists International. Official Methods of Analysis, Method 2011.19 and 993.14. Gaithersburg, MD, USA;**
>
> **52. Pacquette LH, Szabo A, Thompson JJ. Simultaneous determination of chromium, selenium, and molybdenum in nutritional products by inductively coupled plasma/mass spectrometry: Single-laboratory validation. J AOAC Int. 2011;94(4).**

Line 241: Data reporting and analyses

The contents should have been better be placed under "Results" section starting from section 286.

This section is about how the data were analyzed, e.g., statistical methods, so it belongs in Methods, not Results.

Why report in ppb instead of ppm? No wonder the metals were below the detectable limit leading to erroneous conclusion that the vegetables are safe (irrespective of whether cultivated in the urban or rural area (see abstract; line 41).

Study conclusions were not affected by decisions to use ppm or ppb. At no point in the manuscript do we refer to the kale as "safe" and there were no errors, to our knowledge, in comparing metals concentrations in this study to public health guidelines.

Line 255- there is a formula for conversion from fresh weight to dry weight- why not just dry the vegetables at a prescribed temperature till the weight is stable?

We present analyte concentrations in both fresh and dry weight. To do what the reviewer is proposing, we would have had to run of the analyses in fresh weight, dry another set of kale samples, then run the analyses again.

Line 286 Results

Line 287 Kale yields

In this section, reference is made of field samples and market samples – from materials and method section, such categorization was not evident. Can this be elaborated?

Lines 164-166 in the methods clearly explain field samples:

> **"After at least one farmer notified the study team that their kale was ready to harvest (approximately six to seven weeks after transplanting), four kale samples were collected directly from each farm ("field samples") between 11-18 October 2019."**

Lines 174-176 in the methods clearly explain market samples:

> **"…two samples of kale from each farm were collected from the point of distribution ("market samples"), i.e., farmers market, mobile market, or donation site. Farmers obtained six bunches of market-quality kale, which were then randomly assigned to two composite samples of three bunches each."**

Table 1; These are raw data that have not been analysed and hence there is no point of presenting in this section.

Table 1 is a summary of the number of urban and rural farms, and the number of field and market samples per farm. This information is very relevant to the study and should be included for transparency and clarity.

Line 301: Farm history and growing practices

The section does fit under results – moreover, this would only be important if that history led to differences in factors like pH, organic carbon and nutrients as these would affect the availability of these elements to the crop. Can the authors explain the relevance of this section as far as the presentation of the results are concerned

We agree that soil analyses would have been informative, but were outside the scope of a small pilot study. This section reflects the results of the farmer surveys. We believe the information gleaned from the surveys provides relevant background and context about site history, soil amendments, pesticide use, irrigation practices, and other factors that may be relevant to

nutrient and metals concentrations in the kale grown at these sites. Soil samples also would not tell us everything readers might want to know; irrigation frequency, for example, could impact the nutrient density of kale (1).

1. Pathirana I, Thavarajah P, Siva N, Wickramasinghe ANK, Smith P, Thavarajah D. Moisture deficit effects on kale (Brassica oleracea L. var. acephala) biomass, mineral, and low molecular weight carbohydrate concentrations. *Sci Hortic*. 2017 Dec 19;226:216–22.

Line 338; Kale sample processing and storage

This section does not belong to the "results" section. Is there any reason as to why this section is presented under "results" section?

This section describes results from farmer surveys, i.e., time from kale harvest to processing, and use of refrigeration by farmers. The previous header did not clearly reflect this, so we revised the section header to "Time from kale harvest to processing, use of refrigeration".

Line 348: Analytical quality control

The authors present quite interesting results on different types of carotenoids. Unfortunately such differentiations/distinctions were not presented under "materials and methods' section.

All of the types of carotenoids referenced in the Results are also listed in the Methods, starting at line 230:

> **"For carotenoids (alpha-carotene, beta-carotene, gamma-carotene, alpha-cryptoxanthin, beta-cryptoxanthin, lutein, lycopene, and zeaxanthin), samples were extracted and analyzed as previously described using high-performance liquid chromatography (HPLC) for quantitation (40)."**

Table 2: the heavy and essential elements are reported in terms of mg/100mg fresh weights. It is not common to report metal concentrations in fresh weights, instead dry weight is used. Any reasons for this exception?

We have revised the Methods to provide our rationale for this decision, as follows:

> **"Analyte concentrations were reported by laboratories on a per fresh weight basis, e.g., mg vitamin C per 100 g fresh kale. Results herein are also reported on a per fresh weight basis, since this is the form in which kale is typically consumed, i.e., not dehydrated. In the Supporting Information and Supporting Data Tables we additionally present results after converting to dry weight, to control for moisture content."**

In our experience, preferences for fresh vs. dry weight results vary depending on the scientific discipline of the reader, so we provided both.

And for the case when the concentration is in ppm fresh weight – why not mg/kg since we are dealing with solid particles and not liquid?

There is precedent for using ppm/ppb when describing metals concentrations in foods. For example, the US Food and Drug Administration's Total Diet Study uses ppb when describing metals concentrations in foods. https://www.fda.gov/media/159745/download

Note that here the reporting is in ppm yet in the materials and method the authors always made reference of ppb.

We chose ppm or ppb depending on context. In the methods we are referring to detection limits, which were very low: "5, 10, and 50 ppb for lead and uranium, vanadium and arsenic, and nickel, respectively." Using ppm in this context would require a cumbersome format for the reader, whereas the values reported in the results are larger, and thus better suited for ppm, with the exception of uranium which is present is much lower concentrations.

There is a column for "current mean- Urban" and "current mean, rural" – were these results analysed? Please use t-test to compare the two. But since the mean for urban had can only two replications, is it possible to have calculation using two replications?

To compare urban and rural samples, we used a two-sided Mann-Whitney U test, which is the non-parametric version of the t-test. This is described in lines 288-295 of the Methods. The results of tests are shown for each analyte in Figures 2 and 3. It is possible to run the test with a sample size of six (four rural and two urban farms), although this reduces the level of statistical significance.

In the figures, the symbols U1, U2, R1, R2, R3 and R4 are used yet there was no mention of these under "materials and method" section. It would have been polite for the authors to have figure captions where all these could be explained. Otherwise the way they are, it is not possible to interpret.

This is already explained in the Methods, line 136. We have also added this text to the caption for Table 1:

> **"For reporting results, farms were anonymized by assigning a unique identifier beginning with "U" for urban farms and "R" for rural farms."**

The captions for Figures 1 through 3 already include the following:

> **"Site identifiers (x-axis) with "U" represent urban farms, identifiers with "R" represent rural farms."**

It is quite interesting that almost all the results show p-values more than 0.05 hence not significant. Is there possible reason? Could it be that calculation was not well done?

The small sample size (N=6) made it impossible to obtain P values below 0.05. This was explained in the results and discussion, but we have revised the manuscript to make the explanation clearer and more prominent, as follows:

> **Lines 433-437: "Since we had to use mean values for Mann-Whitney U tests, sample sizes (N=6 mean values for each analyte, one per farm) were too small to produce statistically significant results from urban-rural comparisons…"**
>
> **Lines 451-455: "Small sample size (N=6 mean values for each analyte, one per farm) precluded the possibility of statistically significant differences in analyte concentrations between urban and rural field samples. However, P values were as low as 0.06 for arsenic and 0.11 for all carotenoids and vitamins, copper, iron, potassium, zinc, lead, and vanadium (Figs 2-3)."**
>
> **Lines 614-616: "Although small sample size (N=6 mean values for each analyte, one per farm) precluded the possibility of statistically significant differences between urban and rural field samples…"**

Discussion

Since the methodology needs serious reworking (a lot need to be cut out – especially the survey part and only remain with experimental components). And once the result section is more comprehensive and clear (cut out on survey and reduce the subsections under results; same with discussion). Once there is that kindly harmony, it is too premature to take a look at the discussion.

The surveys were a relevant part of the study design, as they allowed us to gather data about farm characteristics, growing practices, time between harvest and distribution at markets, and other information that could help explain observed nutrient and metals concentrations and provide context for the results.

Reviewer #5

The material and method section is too narrative, and contains a lot of redundant information. It should be more concise and clear. E.g. Line 135 Redundant information. Line 162 the same.

Re: Line 159: "After harvesting, a follow-up survey was emailed to the representative at each farm." This is the first and only time the follow-up survey is described, so we are not clear why the reviewer felt it was redundant.

Re: Line 186, sample processing: "Upon arrival at the BSPH laboratory, each sample was inspected by the study team. Leaves deemed edible by the study team (e.g., not yellow or covered in pests) were separated from the central stem and roots…" This is the first and only time this step is described, so we are not clear why the reviewer felt it was redundant.

Why authors choose different digestion methods for essential vs trace elements analysis?

Standard methods, validated for the elements analyzed, were used for each element profile (nutritional elements and metals). Different digests were used simply for convenience based on the timing of analysis.

Lines 516-519 already mentioned, not part of the discussion

We have deleted this paragraph.

Reviewer #6

In the manuscript entitled "Nutrients and Non-Essential Metals in Darkibor Kale Grown at Urban and Rural Farms: A Pilot Study", the authors studied a pilot plant to explore potential differences in nutritional elements, non-essential elements, and some health-relevant nutrients in kale grown at urban versus rural farms. The manuscript is very relevant and interesting. However, the authors need to clarify some parts before publication on Plos one.

The following are my comments and suggestions:

General comments:

The authors need to revise all the units and the effective numbers in the whole manuscript.

It is not clear what sort of revisions the reviewer is asking for. In the text, figures, and tables we provide detailed units (e.g., mg calcium per 100g fresh weight kale) for all quantitative statistics.

Abstract:

The abstract needs to contain all parts of the manuscript. So the authors need to improve this topic, including parts of the methodology. The authors need to insert some results (numbers) , for example, when the authors said "Concentrations of elements were generally higher in kale from urban farms", how much higher? A conclusion sentence about their work is necessary too.

We have significantly expanded the abstract to include details about the methodology, quantitative statistics, and several concluding statements about key conclusions:

> **"Kale is a nutrient-dense leafy vegetable associated with wide-ranging health benefits. It is tolerant of drought and temperature fluctuations, and could thus serve an increasingly important role in providing a safe and nutritious food supply during the climate crisis, while kale's ease of cultivation and ability to be grown in a wide range of soils make it a good fit for urban agriculture. In this pilot study we explored potential differences between kale grown at urban versus rural farms. We planted kale seedlings (Darkibor variety) at three urban and four rural farms in and around Baltimore City, Maryland, instructed farmers to cultivate them using their usual growing practices,**

harvested the kale from fields and points of distribution, and analyzed it for concentrations of carotenoids, vitamins C and K_1, ten nutritional elements, and eight non-essential metals. Although small sample sizes precluded statistical significance for some analyses, we identified non-significant but potentially meaningful differences in concentrations of several components between urban and rural kale samples. Compared to urban samples, mean concentrations of carotenoids and vitamins were 22-38% higher in rural field samples. By contrast, mean concentrations for eight nutritional elements were higher in urban field samples by as much as 413% for iron. Compared to rural field samples, mean concentrations of nine non-essential metals were higher in urban samples, although lead and cadmium concentrations for all samples were below public health guidelines. Some urban-rural differences were more pronounced than those identified in prior research. For six elements, variance within urban and rural farms was greater than variance between urban and rural farms, suggesting urbanicity may not be the primary driver of some observed differences. For some nutrients, mean concentrations were higher than upper ranges reported in prior estimates, suggesting kale may have the potential to be more nutrient-dense than previously estimated. The nutritive and metals composition of this important crop, and the factors that influence it, merit continued investigation given its growing popularity."

Introduction:

The introduction is interesting and contextualizes the environmental problem for the reader. However, the authors should insert more recent references in the Introduction and it´s essential to describe the potentially toxic metal levels allowed in the national and international legislation. Also, when the authors said on page 4 lines 83-84"concentrations of essential and non essential (arsenic (As), barium (Ba), cadmium (Cd), chromium (Cr), lead (Pb)) metals in.." trivalente chromium is essential for the human being. The authors need to research more specific references about it. Please, pay attential to this part including about chemistry speciation.

The reviewer makes an excellent point about chromium. We have revised the manuscript as follows to acknowledge this:

We added the following to lines 214-215 of the methods, and the captions for Table 2 and Figure 3: "Chromium was not speciated between hexavalent chromium (a carcinogen) and trivalent chromium (an essential nutrient)."

We added the following to the discussion, starting at line 530: "…levels of chromium were not speciated between hexavalent chromium (a carcinogen) and trivalent chromium (an essential nutrient), thus if the levels in kale were predominantly comprised of the latter there would be little cause for concern…"

Re: international standards, FAO/WHO maximum recommended levels for lead (0.3 ppm) and cadmium (0.2 ppm) are displayed in Figure 3 and discussed in the manuscript as follows:

Lines 464-467 of the results: "…lead and cadmium concentrations for all samples, including urban (mean Pb: 0.13 ppm, mead Cd: 0.03 ppm) and rural (mean Pb: 0.01 ppm, mean Cd: 0.02 ppm) field samples, were below the maximum levels (Pb: 0.3 ppm,

> **Cd: 0.2 ppm; Fig 3) specified by the Food and Agriculture Organization of the United Nations (FAO) and the World Health Organization (WHO) (52).”**

> **Line 568-570 in the discussion: “…concentrations of lead in this study were below FAO/WHO standards (52) (Fig 3; cadmium levels were well below the standard, and there are no FAO/WHO standards for other metals at the time of writing)…”**

Page 5: “Building upon the existing body of evidence..” The authors need to write more about the aim of the work. They determined a lot of importance parameters obtaining different kind of results, but what is the main question to be answered?

We have revised this paragraph to provide more detail about the study aims, as follows:

> **“Building upon the existing body of evidence, we conducted a pilot study to assess levels of select health-relevant nutrients, nutritional elements, and non-essential metals in kale grown at two urban and four rural farms; and to gather data about site history, farming practices, environmental conditions, and other factors that might influence the properties of kale grown at those sites. The aim of the study was to explore the degree to which concentrations of these analytes might differ between urban and rural farms, and possible reasons for those differences. The analytes of interest were…”**

Materials and methods:

Page 06: The authors need to standardize the date. Sometimes the authors write day, month and year and other times no.

We added the year to line 147: “Between 27-29 August 2019…”

Page 10: The authors need to explain more about the digestion process, including more information as the weight of the samples, temperature, final volume, acid concentration, etc.

Standard methods, validated for the elements analyzed, were used for each element profile (nutritional elements and metals). Different digests were used simply for convenience based on the timing of analysis.

All of the methods used have been previously reported in the references cited (including different citations for the nutritional elements and metals), so details are not repeated and only a brief description of methodology is noted.

For the sake of brevity, citations are already provided for lines 236-240 in lieu of repeating all of the methods in detail:

> **Nine elements (Ca, Cu, Fe, K, Na, Mn, Mg, P, and Zn) were analyzed by inductively coupled plasma emission spectroscopy (ICP) after digestion with concentrated hydrochloric acid (42). The remaining elements (As, Ba, Cd, Cr, Pb, Mo, Ni, U, V) were**

analyzed by ICP with mass spectrometry detection (ICP-MS) after digestion with concentrated nitric acid and water using a closed-vessel microwave digestion system (43,44).

42. Association of Official Analytical Chemists International. Official Methods of Analysis, Method 984.27, 985.01,and 2011.14. Gaithersburg, MD, USA;

43. Association of Official Analytical Chemists International. Official Methods of Analysis, Method 2011.19 and 993.14. Gaithersburg, MD, USA;

44. Pacquette LH, Szabo A, Thompson JJ. Simultaneous determination of chromium, selenium, and molybdenum in nutritional products by inductively coupled plasma/mass spectrometry: Single-laboratory validation. J AOAC Int. 2011;94(4).

Page 10: The authors need to write more about organic and inorganic determination as lines of emissions, analytical concentration curve, detection and quantification limits. The authors need to mention the replicates here too.

Refer to responses to previous comments: The details of methodology are not repeated here since they are standard/published methods for which details are given in the references cited. Replicates, and validation of the present analyses using certified reference materials is described in Methods (lines 241-254) and Results (lines 381-391 and Table S1)

Replicates ("duplicates") are described in the following section starting at line 241, "Analytical quality control."

Detection limits are discussed at lines 271-276 under "Data reporting and analysis." Specific samples that were below detection limits are reported in Table S2.

Page 12, line 255: The authors need to include the reference for this equation.

We have added a reference (US EPA Exposure Factors Handbook), and revised the paragraph preceding the equation to provide more detail and context:

Analyte concentrations were reported by laboratories on a per fresh weight basis, e.g., mg vitamin C per 100 g fresh kale. Results herein are also reported on a per fresh weight basis, since this is the form in which kale is typically consumed, i.e., not dehydrated. In the Supporting Information and Supporting Data Tables we additionally present results after converting to dry weight, to control for moisture content. Fresh weight concentrations were converted to dry weight using the following formula adapted from the US Environmental Protection Agency (EPA) (48):

$$\boldsymbol{C_{a,i,dry} = \frac{C_{a,i,fresh}}{1 - W_i}}$$

Where *C* is the concentration of analyte *a* in sample *i* and *W* is the wet fraction for sample *i*.

Results and discussion

In general, the authors need to discuss more the results comparing with the literature. The authors need to explore further the parts containing discussion and results, for example, in the topic "Correlations among analytes", the authors said "We also observed some statistically significant relationships among analyte concentrations and other properties of the kale samples.", the authors need to discuss and to say more specifically about all the parameters studied. In the sentence, "Similarly, concentrations of elements and metals were in some cases correlated with one another. ", the authors need to specify the elements here. What´s the diference between metals and elements?

Re: "We also observed some statistically significant relationships among analyte concentrations and other properties of the kale samples," these are detailed in the Results and Fig 4. For the sake of brevity we didn't repeat all of the specific findings in the Discussion, but we do include an example: "The ratio of leaf mass to stem mass, for example, was negatively correlated with some carotenoids and vitamin K_1, and positively correlated with some nutritional elements and non-essential metals," followed by discussion: "Further research could shed light on the reasons for these relationships; studies have shown that some metals, for example, tend to accumulate at higher levels in different parts of a plant, e.g., roots vs. leaves (60–62)."

We agree that "elements and metals" is confusing; we have revised this to "nutritional elements and non-essential metals." The specific elements are detailed in the Results and figures.
